# The de novo FAIRification process of a registry for vascular anomalies

**DOI:** 10.1186/s13023-021-02004-y

**Published:** 2021-09-04

**Authors:** Karlijn H. J. Groenen, Annika Jacobsen, Martijn G. Kersloot, Bruna dos Santos Vieira, Esther van Enckevort, Rajaram Kaliyaperumal, Derk L. Arts, Peter A. C. ‘t Hoen, Ronald Cornet, Marco Roos, Leo Schultze Kool

**Affiliations:** 1 Department of Medical Imaging, Radboud Institute for Health Sciences, Radboud University Medical Center, Nijmegen, The Netherlands; 2 Department of Human Genetics, Leiden University Medical Center, Leiden, The Netherlands; 3 Department of Medical Informatics, Amsterdam Public Health Research Institute, Amsterdam UMC, University of Amsterdam, Amsterdam, The Netherlands; 4 Castor EDC, Amsterdam, The Netherlands; 5 Center for Molecular and Biomolecular Informatics, Radboud Institute for Molecular Life Sciences, Radboud University Medical Center, Nijmegen, The Netherlands; 6 University Medical Center Groningen, Department of Genetics and Genomic Coordination Center, University of Groningen, Groningen, The Netherlands

**Keywords:** Rare diseases, Patient registry, Vascular anomalies, FAIR data, FAIRification process, Interoperability

## Abstract

**Background:**

Patient data registries that are FAIR—Findable, Accessible, Interoperable, and Reusable for humans and computers—facilitate research across multiple resources. This is particularly relevant to rare diseases, where data often are scarce and scattered. Specific research questions can be asked across FAIR rare disease registries and other FAIR resources without physically combining the data. Further, FAIR implies well-defined, transparent access conditions, which supports making sensitive data as open as possible and as closed as necessary.

**Results:**

We successfully developed and implemented a process of making a rare disease registry for vascular anomalies FAIR from its conception—de novo. Here, we describe the five phases of this process in detail: (i) pre-FAIRification, (ii) facilitating FAIRification, (iii) data collection, (iv) generating FAIR data in real-time, and (v) using FAIR data. This includes the creation of an electronic case report form and a semantic data model of the elements to be collected (in this case: the “Set of Common Data Elements for Rare Disease Registration” released by the European Commission), and the technical implementation of automatic, real-time data FAIRification in an Electronic Data Capture system. Further, we describe how we contribute to the four facets of FAIR, and how our FAIRification process can be reused by other registries.

**Conclusions:**

In conclusion, a detailed de novo FAIRification process of a registry for vascular anomalies is described. To a large extent, the process may be reused by other rare disease registries, and we envision this work to be a substantial contribution to an ecosystem of FAIR rare disease resources.

**Supplementary Information:**

The online version contains supplementary material available at 10.1186/s13023-021-02004-y.

## Background

Rare disease (RD) registries contain valuable information for improving diagnosis, treatment and event prevention [1]. For this reason, extensive research has been performed on setting up high quality and effective RD registries [2, 3]. Using this information for research generally requires data from more than one registry, due to the low prevalence of RDs. However, RD registries are distributed across the world. Also, data from these registries are available in heterogeneous formats and multiple languages. As a consequence, optimising the use of RD registries for research requires substantial effort, and is further complicated by legal constraints and the need for proper precautions for protecting the privacy of the sensitive data. Kodra et al. [2] and Rubinstein et al. [4] mention the FAIR data principles as a means to make the use of distributed RD data as effective as possible.

The FAIR data principles aim to enable efficient analysis of data across multiple sources through enhancing their Findability, Accessibility, Interoperability and Reusability for humans and computers [5]. Data that are FAIR at their source are prepared for efficient computational analysis across multiple FAIR sources. For instance, multiple FAIR sources can be queried simultaneously to answer a research question in so-called ‘federated queries’ that do not require source data to be moved to one central location [6]. FAIR data are not open by definition. FAIR implies well-defined, transparent access conditions, which supports making data as open as possible and as closed as necessary [7]. By applying the FAIR principles to RD registries (here referred to as the data collected from RD patients), analysis across multiple RD registries and other relevant FAIR data is made possible, even when access criteria differ per source.

The added value of the FAIR principles for RD research led to early acknowledgement by the RD community, and in 2017 the FAIR principles became a recognised resource by the International Rare Disease Research Consortium (IRDiRC) [8]. For example, since 2014, “Bring Your Own Data” workshops (BYODs) have been held to accelerate the adoption of the FAIR principles [9–11]. This includes a series of annually recurring BYODs in the RD domain. Over the years, the FAIRification process applied in BYODs has been explored and refined, and finally described step-by-step in a generic workflow [12]. Other research communities have also developed similar workflows, such as the workflow for FAIRification of data for health research by Sinaci et al. [13]. An important step of this FAIRification process is to make data interoperable and machine-readable in a format that can be read and processed by computers. Data and data access protocols can be made machine-readable by annotating and structuring them with ontologies, which also ensures that data may be more easily analysed across RD registries using federated queries. IRDiRC has recognised ontologies to describe e.g. phenotypes (Human Phenotype Ontology—HPO [14]) and rare diseases (Orphanet Ontology for Rare Disease—ORDO [15]).

Another effort to further improve research across RD registries is the "Set of Common Data Elements for Rare Diseases Registration" (CDEs) released by the Joint Research Centre of the European Commission [16]. The set consists of 16 data elements that are considered to be essential for research. Next to this, the European Commission has set up the European Rare Disease Registry Infrastructure (ERDRI) to facilitate findability of RD registries [17], an important task for the European Reference Networks (ERNs) [18]. The ERNs are virtual networks at a European level, involving healthcare institutes recognised as expert centres for specific RDs. ERNs aim to facilitate discussion on complex or rare diseases and conditions that require highly specialised treatment. Also, they aim to concentrate knowledge and resources. To that end, ERNs have been provided funding to set up registries [19]. Minimum requirements include support for the CDEs, linking registries and making them interoperable. The European Joint Programme on Rare Diseases (EJP RD) further supports registries in implementing the FAIR principles. VASCERN is the ERN focusing on rare multisystemic vascular diseases [20]. VASCERN is subdivided into thematic working groups, one of which is the Vascular Anomalies working group, VASCA [21]. VASCA includes nine centres with individual databases and data collection processes.

Here, we describe how we set up a FAIR registry for vascular anomalies (hereafter referred to as the VASCA registry) in one of the VASCA centres, Radboud university medical center. The objectives were to (1) base our VASCA registry on the CDEs and the FAIR principles to enable it for analysis across RD registries, and (2) implement de novo FAIRification in our VASCA registry, where data are made FAIR automatically and in real-time upon collection. By doing all the hands-on work for the FAIRification before data collection, data is made FAIR through entering them into an Electronic Data Capture (EDC) system. This mitigates the need for post-hoc FAIRification operations, which include repeated, semi-manual conversions of the data collected into machine-readable data that is performed after data collection. The de novo approach saves time and budget for the actual FAIRification of the data in the VASCA registry. To our knowledge, this is the first attempt to create a de novo FAIR RD registry, and may therefore serve as an example for (and be reused by) other registries. This article focuses on the FAIR part of the registry and not on setting up a registry in general (for recommendations for setting up effective and high quality RD registries in general see for example Kodra et al. [2] and Stanimirovic et al. [3]). Therefore, this article describes the complete de novo FAIRification workflow, from identifying FAIRification objectives and required expertise to querying data over a FAIR Data Point. The technical implementation in the EDC system is described in detail in Kersloot et al. [22].

## Results

**Fig. 1:**
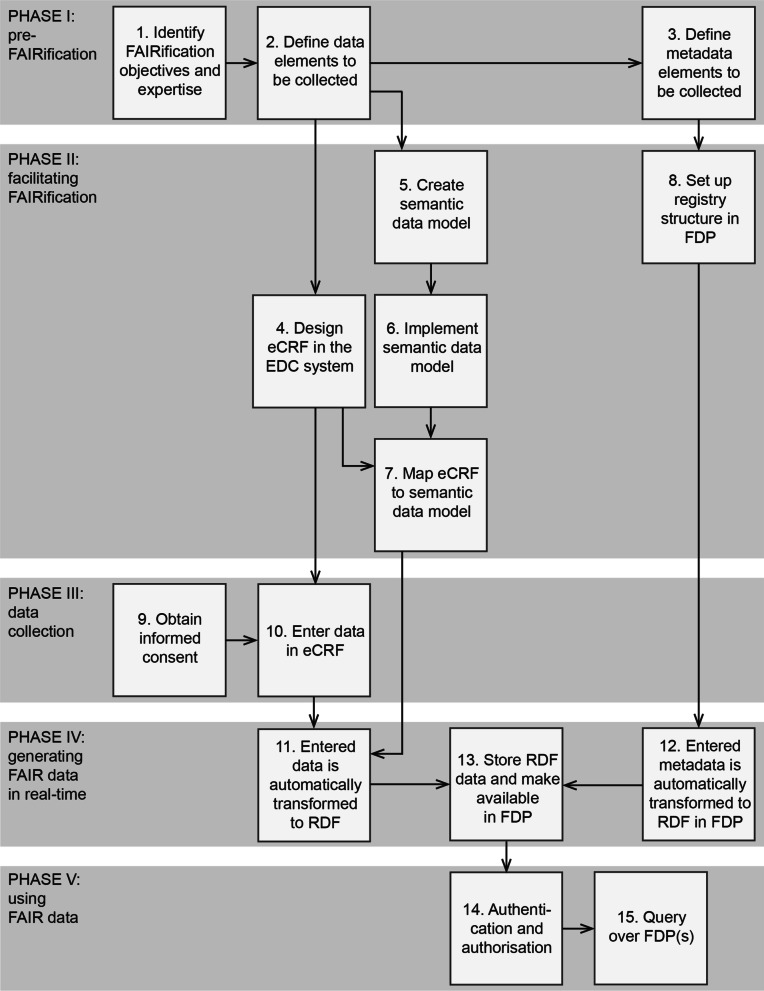
Workflow of the de novo FAIRification process of a registry for vascular anomalies. The workflow is divided into five ‘phases’: pre-FAIRification, facilitating FAIRification, data collection, generating FAIR data in real-time, and using FAIR data. The phases are further specified by ‘steps’ indicating practical FAIRification tasks. Abbreviations: electronic Case Report Form (eCRF), Electronic Data Capture (EDC), Resource Description Framework (RDF; machine-readable language), FAIR Data Point (FDP)

We present a workflow for the de novo FAIRification process of the VASCA registry (Fig. 1, see Methods and Additional file 1: Supplementary Methods). In the following sections, we describe how our approach contributes to each of the four facets of FAIR as well as how it can be reused by other RD registries. For an automated assessment on the FAIRness [23] of our output, the FAIRified data and metadata, using the FAIR Evaluation Services [24] see [22].

### Contribution to the four facets of FAIR

#### Contribution to F—Findable

We made the VASCA registry *findable* in searches for humans and computers by providing a description of the registry data (‘metadata’) relating to findability. The metadata was structured and made machine-readable using the Data Catalog Vocabulary (DCAT) standard (see details in Additional file 1: Supplementary Methods—step 8). Each vocabulary term in the DCAT standard has a globally unique identifier with a machine-readable definition that can be found on the internet. The DCAT standard provides terms to denote metadata that facilitates findability such as: database title (‘Registry of Vascular Anomalies’), description (‘Databases of the ERN vascular anomalies’), and country (‘The Netherlands’). The metadata was made available in a FAIR Data Point [25, 26] and represented for humans in a visual interface and for computers in the Resource Description Framework (RDF) [27]: http://purl.org/castor/fdp/catalog/vasca. Note that *finding* the VASCA FAIR Data Point does not necessarily mean that the registry data can be *accessed*, *interoperated* and *reused*. This is covered in the following sections.

VASCA registry metadata was also made findable for humans in the European Directory of Registries (ERDRI.dor) [28] under the namespace: “European Rare Vascular Anomalies Registry”. Metadata in ERDRI.dor include the medical areas involved, rare diseases registered, characterisation of the registry, and affiliation to the ERN. At this time the metadata registered in ERDRI.dor is not yet findable for computers in a FAIR format such as DCAT.

#### Contribution to A—Accessible

We made the VASCA registry *accessible* by providing metadata in the VASCA FAIR Data Point that describes the access protocols for the registry data in the DCAT vocabulary’s ‘distribution’ component within the FAIR Data Point record. Access protocols define where requests for access (calls) are sent and under which conditions a call will be accepted in order to gain access to data. In addition, calls will generally contain a payload that follows a particular interface format in order to be interpreted by the data endpoint. We accept the HTTP protocol for calls to the data endpoint and accept two interface formats—SPARQL Protocol and RDF Query Language (SPARQL) via HTTP GET, and simple HTTP GET. SPARQL allows for the execution of queries on data that is made available in RDF (similar to SQL for data in relational databases) and can also be used for federated querying across multiple data sources. To retrieve data, SPARQL queries use the semantic data model and ontologies that describe the data (see Additional file 1: Supplementary Methods and Figure S2). Another access protocol was included for simple retrieval, to support viewing or exporting the machine-readable data and can, for example, be used to perform analyses on a local computer.

Access to the VASCA registry data is managed by the authentication and authorisation system of the EDC system (see Additional file 1: Supplementary Methods—step 14). Access can be granted to users (currently only humans) by the VASCA registry contact person specified in the metadata in compliance with the informed consent (see Additional file 1: Supplementary Methods—step 9). Note, the ‘Reusability’ facet also relates to the access of registry data and metadata, but focuses on permission and trust, such as consent, license, and attribution.

#### Contribution to I—Interoperable

We made the VASCA registry machine-readable and *interoperable* at a number of levels. First, the metadata was structured using the DCAT vocabulary following the FAIR Data Point specification. This contributes to machines being able to query the existence of the registry and its content descriptions. Second, the registry patient data collected in the eCRF was structured using a semantic data model [29] constructed from terms and relations in commonly used ontologies (e.g. SNOMED CT and the IRDiRC recognised ontologies HPO and ORDO). Third, the VASCA registry was configured to collect data for the CDEs, and descriptions of these data elements were registered in the ERDRI Metadata Repository (ERDRI.mdr) [30] under the namespace:’European Rare Vascular Anomalies Registry’. The CDEs do not directly address any FAIR interoperability principles but do increase the compatibility of data in registries for certain analyses. Using an ontological model to define the meaning of these data elements ensures that we give access to a harmonised set of data elements and facilitate integration of CDEs from different registries, even across different ERNs. We note that without such an ontological model, computers cannot assess that common data elements are indeed common.

#### Contribution to R—Reusable

We made the VASCA registry *reusable* for humans and computers by providing metadata in the VASCA FAIR Data Point relating to reusability. Each metadata layer contains references to a license, the publisher (organisation and person), media type, version, and timestamp of the underlying data or metadata. More metadata is stored in the ERDRI.dor overview, but at this time this information can only be accessed after logging into the EU RD platform and is not yet accessible in a FAIR format. The VASCA registry collects clinical data, which contains privacy sensitive data. By making it FAIR, the registry data is as closed as necessary and as open as possible for other researchers (humans) and computers. The metadata in the VASCA FAIR Data Point is open with a CC0 license [31], whereas, the patient-derived data in the VASCA registry is only accessible to researchers that have been granted access by the registry contact person (see ‘Accessibility’ facet).

The metadata in the VASCA FAIR Data Point contains a reference to an RDF ‘distribution’ of the data that can be queried in terms of the CDE semantic model (see Additional file 1: Supplementary Methods and Figure S2). An example ontological query could be: “List all phenotypes reported for patients diagnosed with any type of vascular anomaly or angioma from VASCA FAIR Data Points in France, Germany, and The Netherlands”. These queries can span multiple databases, as ontologies are not bound to a single dataset, thereby enabling federated querying.

### Reusability of the de novo FAIRification process

Several aspects of the de novo FAIRification process of the VASCA registry have been made available and can be reused by ERNs for setting up their FAIR RD registries that collect the CDEs. The workflow (Fig. 1) and expertise (Additional file 1: Table S1) used in our FAIRification projects can be reused for organisation and preparation of other projects. Likewise, other aspects developed for our project that can be reused are our interpretations, semantic data model, and eCRF of the CDEs, FAIR implementations in the EDC system, and structured metadata describing the VASCA registry.

We interpreted the CDEs in order to define what data should be collected in the registry (Additional file 1: Supplementary Methods—step 2). In our opinion the CDEs are multi-interpretable, hence, downstream implementations depend on these. We therefore properly defined and made our interpretations reusable for others in an extensive manual (available upon request).

We created a semantic data model that describes the CDEs and their relation, and made it available on GitHub [29] (Additional file 1: Supplementary Methods—step 5). Efforts to further develop and maintain the model are taking place [32] (also see Discussion). The goal of sharing the model is twofold: (1) Reuse: other ERNs can directly implement the model and would only need to extend the model with elements that are not a part of the CDEs; (2) Improving interoperability: It is easier to perform analyses across datasets if they use the same semantic model (using different models requires ontology mapping to facilitate federated querying).

Castor EDC [33], the vendor of the EDC system used in our project, developed the technology to facilitate the de novo FAIRification of the VASCA registry (phase ii in Fig. 1). The eCRF designed for the CDEs, including the technology to translate to machine-readable format, are reusable (Additional file 1: Supplementary Methods—steps 6 and 7). The eCRF can be copied directly to a new database within the EDC system, to initiate a new ERN registry. Some ERN-specific adaptations may be necessary. For instance, diagnosis is registered using a drop-down menu focusing on vascular anomalies and should therefore be adjusted for an ERN with a different focus. The ontologies used in the CDE semantic data model are not limited to an area of disease. The developed (eCRF to RDF) data transformation application (Additional file 1: Supplementary Methods—step 6 onwards; [17]) is generic and can be reused by other registries and clinical trials, ensuring that new FAIRification projects can easily be set up within the EDC system. Likewise, other registries in the EDC system can reuse the FAIR Data Point structure and query functionalities developed for the VASCA registry (Additional file 1: Supplementary Methods—steps 8, 12, 13, 14, and 15). Furthermore, we have made our eCRF interoperable and reusable, as the codebook describing the eCRF templates containing the CDEs and the ontologies to annotate them is openly available in ART-DECOR [34]. Via the openly available iCRF Generator tool [35], the codebook can be directly implemented in other EDC systems such as OpenClinica and REDcap.

Finally, structured metadata describing the VASCA registry on ERDRI.mdr and the FAIR Data Point are reusable. Structured record level metadata of the CDEs were included in ERDRI.mdr (name and descriptions of data collected for the CDEs). Other registries can clone and reuse the VASCA ERDRI.mdr metadata if they are setting up a registry according to the CDEs.

## Discussion

This project aimed to (1) base our VASCA registry on the CDEs and FAIR principles to enable it for analysis across RD registries, and (2) implement de novo FAIRification in our VASCA registry, where data are made FAIR automatically and in real-time upon collection. With regard to this first objective, we created an ontology-based semantic model of the CDEs recognised by the European RD community and implemented this model in our eCRF. As a result, machine-readable data can be queried through a FAIR Data Point, thereby facilitating analysis across RD registries. Within this project, we opted for a de novo approach (objective 2). To this end, we developed software that converts ‘normal data’ entered in the eCRF automatically into machine-readable data, thereby following the semantic model implemented. This comes with the great advantage, that data is made FAIR and available for research upon data entry as well as that clinical people are not tasked with the technical data conversion steps.

The step-by-step description provided in this paper, might help other ERNs and RD stakeholders setting up their own FAIR registries. In the following sections, we discuss the lessons we learned during the project and describe our ideas for future developments.

### Lessons learned

#### The interpretation and collection of the Common Data Elements

The CDEs include seemingly simple elements that turned out to be multi-interpretable. As an example, ‘sex’ can be interpreted as both genotypic sex and declared sex. Or, the element ‘date of first contact with a specialised centre’ requires a clear definition of a specialised centre; should it be a Healthcare Provider (HCP) that is a full ERN member, or can it also be an expert unit not being part of the ERN yet? In order to use a registry for research it is essential that it is clearly defined how the CDEs are interpreted for each registry to avoid the possibly false assumption that they are interpreted uniformly across registries. We recommend that all registries clearly document their interpretations of the CDEs, for instance in a manual such as the one created for our VASCA registry. Ideally, guidelines are provided on a European level.

Another issue regarding the CDEs is the discrepancy between data to be collected for the registry and data that is actually collected within the Electronic Health Record (EHR) in daily clinical practise. For example, the ORPHAcodes used to define the diagnosis are very extensive and include a hierarchy. In clinical practice, clinicians may not use ORPHAcodes to code diagnoses in a patient’s medical record, nor use these detailed categories. Another example is the CDE ‘disability’. The EU prescribes to operationalize the CDE ‘disability’ using the WHO Disability Assessment Schedule (WHODAS). WHODAS, however, is only validated for adults, whereas a significant part of patients suffering from rare diseases are children.

Furthermore, the CDEs form a static description, thereby not capturing changes in the patients’ situation over time (follow-up). The data collected for the CDEs only represent their situation at the moment of data capture, but for some CDEs changes over time are likely to happen. For example, the execution of (new) diagnostic tests in a specialised centre or starting (new) treatments might very well affect the outcome of the disability score. Also, over time, new test results might become available (e.g. genetic tests, imaging), affecting the diagnosis. It is currently unclear in what cases and within what timeframe the information for already included patients should be updated. To this end, advice and alignment on when to assess and update the CDE data is needed.

The 16 CDEs form the core of the registries, but based on discussions with clinicians across Europe, we concluded that clinicians wish to extend the dataset with disease-specific elements that most probably differ between registries. This is, however, something that affects the work required for FAIRification, as the semantic data model should be extended with these disease-specific elements. Consequently, guidelines are required for extending the core CDE model with disease-specific elements. Also, coordination on data modelling is required between ERNs and/or registries to ensure compatible solutions (see also next section).

#### The semantic data model of the Common Data Elements

We learned that selecting ontologies can be difficult, as this process depends on the interpretation of the CDEs. When a CDE is interpreted similarly in different projects, it is recommended that the same ontology is used, as this prevents the need for mapping between ontologies. To this end, we recommend that a standard set of ontologies should be defined for ERN registries (in addition to HPO and ORDO) to enhance interoperability. When a CDE is interpreted differently in different projects, correct interpretation by FAIR should be facilitated: differences in interpretation are acceptable as long as these interpretations are explicit and represented in both human- and machine-readable formats.

In the current project, interpreting the CDEs and selecting the corresponding ontologies were handled as two distinct activities and to some degree performed separately and independently. As shown in Additional file 1: Table S1, different expertises were required for interpretation of the data elements (clinicians specialised in and patient advocate for vascular anomalies) and generating a semantic data model (local and FAIR data steward, semantic data modelling specialist and clinicians specialised in vascular anomalies). To enhance efficiency and quality of the semantic data model, we recommend both expertise to be at the table when developing and discussing the semantic data model (at least in the conceptual modelling part).

During our FAIRification project, as expected, the semantic data model continued to evolve. We documented and implemented the first complete version of the model. Currently, the model is being further developed and optimised by ontology experts in EJP RD. Besides this, in future we foresee ongoing adjustments due to e.g. improvement of technologies, ontologies, as well as changes to the CDEs themselves. The question is if, how, and to what extent this would affect the interoperability of datasets. Therefore, one should think of how the community should deal with the use of different models (versions). Researchers should be able to use different versions of the model. Therefore, mapping between versions is essential. We foresee different approaches to deal with this. One would be that the ‘owner’ of the registry adjusts to a new model or new version. Another approach would be that newly developed models or versions are made mappable to earlier versions, meaning that the community should be provided with either mapping tools or mappable models when the CDE-based semantic data model is further optimised. We would argue that the latter approach would be preferred as it requires less effort of the end users. Particularly if many researchers (end users) make use of the same model, this second approach is beneficial, as the modelling work only needs to be done once. In contrast, in the first approach all users would need to adjust to the model individually. Further optimisation of the model also leads to further complexities such as different versions of semantic models needing to be mapped to different versions of the eCRF. In both approaches, our de novo FAIRification framework implies less extra work when a model is changed compared to post-hoc FAIRification. The conversion into a machine-readable format is more or less automatic and would only require implementing the updated model in the eCRF (Methods step 6). In contrast, post-hoc FAIRification would require additional redoing the semi-manual conversion into a machine-readable format.

#### FAIR implementation in the EDC system

Enabling de novo FAIRification in the Castor EDC system required developing the necessary technology from scratch. We first piloted the generation of machine-readable data to test the integration between the data transformation application and the EDC system. We prioritised developing a generic tool, rather than a smaller registry-specific tool, as it can be used by a large number of registries and clinical studies. The scalability of our approach contributes to making more FAIR data available for the community.

In addition, we decided to implement authentication and authorisation layers in the FAIR Data Point by reusing the authentication and authorisation of the EDC system. This means that at the moment, researchers that do not have access to the database in the EDC system are not able to access the data through the FAIR Data Point.

#### Informed consent

Informed consent is usually required for collecting prospective patient data for scientific purposes. The European Commission has provided the ERNs with a standard patient information folder (PIF) and broad informed consent form (ICF). Our Institutional Ethical Review Board did not approve the PIF and ICF for scientific registries. Main reasons were that the information provided on data handling was too limited. Therefore, our Institutional Ethical Review Board requested us to redraw the PIFs and ICFs. This has several possible consequences. Not only do the different centres need to follow local guidelines, one also needs to make sure data exchange is facilitated in an easy way. Future collaborations including data sharing with other parties and the own ERN working group should explicitly be part of the PIFs and ICFs.

#### Preconditions for an effective (FAIR) registry

Previous research has investigated the preconditions for the establishment of a RD registry. Using focus group sessions, Stanimirovic et al. [3] identified that the effective development of a national RD registry, followed by the establishment of a RD ecosystem, requires a broad approach that entails a whole series of systemic changes and considerations. Moreover, well-orchestrated and well-funded efforts to achieve this goal should involve coordinated action of all stakeholders, including a regulatory framework, quality design, and enactment of a general RD policy, as well as the alignment of medical, organizational, and technological aspects in accordance with the long-term public healthcare objectives. Most of these aspects are also identified by Kodra et al. [2]. All these prerequisites are also essential for setting up effective FAIR registries. Adding the FAIR aspects to a registry, puts extra ‘pressure’ on several of these preconditions. First, additional demands are made on the IT infrastructure, as it should also facilitate the conversion of clinical data into ontological (meta)data and federated querying via FAIR Data Points. In case of the latter, the FAIR Data Points should be able to connect different (types of) registries. These additional demands on IT infrastructure apply to both development or setting up of the registry and long-term maintenance of the registry. Secondly, the legal basis might be more complex, as there should not only be a legal basis for collecting data, but also for (automated) sharing and re-using data (by others). In case others aim to re-use the data via SPARQL queries in the FAIR Data Point, one should determine if the nature of the query and purpose for which the query results will be used, match the original legal basis of the registry. Ideally, these aspects are checked automatically in the FAIR Data Point. This technology is yet to be developed. Furthermore, FAIR data stewards, semantic modelling specialists, interoperability experts, and experts on standards for automated access protocols and privacy preservation should be added to the already highly interdisciplinary group of professionals tasked with setting up the registry.

### Future developments

The rapid development of FAIR technologies and possibilities requires us to continuously improve our FAIRification workflow. We are currently working on several aspects, discussed below.

The European Patient Identity Management (EUPID) pseudonymization tool [36] is recommended by the European Commission [37] and aims to ensure that different registries can be mapped on a patient-to-patient level. However, at the time of setting up the VASCA registry, EUPID was not up and running yet and, therefore, not implemented in the VASCA registry. We are currently exploring the technical options to integrate EUPID into the registry, taking aspects related to automation, security, privacy and efficacy into account.

As described in Additional file 1: Supplementary Methods, we mapped the International Society for the Study of Vascular Anomalies (ISSVA) terms to the ORPHAcodes. However, the ISSVA terms not present in ORDO lacked a unique identifier. To comply with the interoperability principles, we are currently transforming the ISSVA classification into an ontology (OWL format), keeping the structure and adding all possible concepts and terms mappings to HPO, ICD, SNOMED CT, ORDO and NCIT. This way, in case an ISSVA term is not present in other existing ontologies, it has a unique identifier.

Setting up a registry requires a good balance between the amount of information one would like to collect, and the amount clinicians are able to provide given the limited time they can spend for each patient. In the current registry, clinicians provide all information. We are currently looking into the possibilities for a patient-driven registry. In patient-driven registries, patients fill in (part of the) data themselves rather than the clinician. This way, we would be able to collect more data with less effort. This would additionally enhance the options for collecting longitudinal data (which is not covered by CDEs), for example on quality of life, medication intake or treatments, thereby allowing additional research questions to be answered.

In addition, reaching interoperability, and thereby facilitating secondary use of data from the EHR, requires the use of ontologies during data collection. Currently, this means that the data from the EHR (both structured fields and notes made by clinicians) should be ‘converted’ into terms used in the ontologies. This is currently mostly manual work and is heavily dependent on interpretation by the person carrying out the data entry in the EDC system. To further optimise and automate this process, we are currently exploring whether software tools that automatically map free text to ontologies can aid in this. An example implementation would be the facilitated mapping of diagnoses extracted from the EHR to HPO or other ontology terms, using software, such as Phenotips [38], Zooma [39], and SORTA [40, 41]. Alternatively, it would be interesting to work on a tool for mapping eCRF data with ontology terms.

The web-based query method in the EDC system can currently only be used to query data in one registry, but work is being done to support querying over multiple registries. This would allow for easier retrieval of relevant information from multiple registries. For further interoperability, we would require an interface that facilitates queries over multiple registries, independent of the EDC system used for construction of the registries.

Next steps will also include the development of human and machine-readable access conditions to the data and, subsequently, the implementation of a mechanism for requesting and granting access to the data.

## Conclusion

In conclusion, we successfully set up a workflow for de novo FAIRification of the CDEs for the registry of vascular anomalies. The methods and lessons learned in the different phases of the FAIRification process are described in detail (for methods see Additional file 1: Supplementary Methods). This may help other ERNs in setting up their FAIR registries.

Next steps are to extend the VASCA registry with disease-specific data elements and to set up this registry in the other VASCA institutes and VASCERN working groups. This will allow us to analyse data across multiple registries using federated queries and, thereby, to demonstrate the added value of making them FAIR.

## Methods

### Workflow of the de novo FAIRification process

The workflow of the de novo FAIRification process for the VASCA registry developed and implemented in this project is divided into five phases: (i) pre-FAIRification, (ii) facilitating FAIRification, (iii) data collection, (iv) generating FAIR data in real-time, and (v) using FAIR data (Fig. 1). The phases are further divided into steps describing practical FAIRification tasks detailed in Additional file 1.

## Supplementary Information

**Additional file 1**:** Supplementary methods**. De novo FAIRification workflow step 1–15.** Table S1**. Expertise required for the FAIRification of a registry for vascular anomalies (VASCA).** Figure S1**. Schematic representation of the generation of machine-readable data in the Resource Description Framework (RDF).** Figure S2**. Metadata layers for the Registry of Vascular Anomalies (VASCA) in Castor EDC’s FAIR Data Point.

## Data Availability

Data sharing is not applicable to this article as no datasets were generated or analysed during the current study. However, (parts of) the methods developed during this project are available online. The manuscript describes what parts of the workflow are available for reuse and where information/ data on the process can be found.

## Abbreviations

API: Application Programming Interface
BYOD: “Bring Your Own Data” workshop
CC0: Creative Common zero
CDEs: Set of Common Data Elements for Rare Disease Registrations
CSV: Comma Separated Value
DCAT: Data Catalog Vocabulary
eCRF: Electronic Case Report Form
EDC: Electronic Data Capture (system)
EHR: Electronic Health Record
EJP RD: European Joint Programme on Rare Diseases
ERDRI: European Rare Disease Registry Infrastructure
ERDRI.dor: European Rare Disease Registry Infrastructure—European Directory of Registries
ERDRI.mdr: European Rare Disease Registry Infrastructure—Metadata Repository
ERN: European Reference Network
EUPID: European Patient Identity Management
FAIR: Findable, Accessible, Interoperable, Reusable
FDP: FAIR Data Point
HPO: Human Phenotype Ontology
ICD: International Classification of Diseases
iCRF Generator: Interoperable Case Report Forms Generator
IRDiRC: International Rare Disease Research Consortium
ISSVA: International Society for the Study of Vascular Anomalies
JSON: JavaScript Object Notation
NCIT: National Cancer Institute Thesaurus
ORDO: Orphanet Ontology for Rare Disease
OWL: Web Ontology Language
RD: Rare disease
RDF: Resource Description Framework
SPARQL: SPARQL Protocol and RDF Query Language
TSV: Tab Separated Values
URL: Uniform Resource Locator
VASCA: Vascular Anomalies
VASCERN: European Reference Network on rare vascular diseases
W3C: World Wide Web Consortium
WHODAS: WHO Disability Assessment Schedule
XML: Extensible Markup Language
