# Supplementary material for: The de novo FAIRification process of a registry for vascular anomalies
Source: Orphanet J Rare Dis. 2021 Sep 4;16:376. doi: 10.1186/s13023-021-02004-y (PMC8418729; doi:10.1186/s13023-021-02004-y)
Supplement: Supplementary file 1 — Additional file 1: Supplementary methods. De novo FAIRification workflow step 1–15. Table S1. Expertise required for the FAIRification of a registry for vascular anomalies (VASCA). Figure S1. Schematic representation of the generation of machine-readable data in the Resource Description Framework (RDF). Figure S2. Metadata layers for the Registry of Vascular Anomalies (VASCA) in Castor EDC’s FAIR Data Point. [file 13023_2021_2004_MOESM1_ESM.docx]

**Supplementary information**

**Additional file 1**

**Supplementary methods**

Phase I: Pre-FAIRification

*Step 1 - Identify FAIRification objectives and expertise*

*Step 2 - Define the data elements to be collected*

*Step 3 - Define the metadata elements to be collected*

Phase II: Facilitating FAIRification

*Step 4 - Design the eCRF in the EDC system*

*Step 5 - Create the semantic data model*

*Step 6 - Implement the semantic data model*

*Step 7 - Map the eCRF structure to the semantic data model*

*Step 8 - Set up registry structure in the FAIR Data Point*

Phase III: Data collection

*Step 9 - Obtain informed consent*

*Step 10 - Enter data in the eCRF*

Phase IV: Generating FAIR data in real-time

*Step 11 - Entered data is automatically transformed to RDF*

*Step 12 - Entered metadata is automatically transformed to RDF in the FAIR Data Point*

*Step 13 - Store RDF data and make it available in the FAIR data point*

Phase V: Using FAIR data

*Step 14 - Authentication and authorisation*

*Step 15 - Query over FAIR data point(s)*

**Supplementary table**

Table S1. Expertise required for the FAIRification of a registry for vascular anomalies (VASCA).

**Supplementary figures**

Figure S1. Schematic representation of the generation of machine-readable data in the Resource Description Framework (RDF).

Figure S2. Metadata layers for the Registry of Vascular Anomalies (VASCA) in Castor EDC’s FAIR Data Point.

**Supplementary methods**

**Phase I: Pre-FAIRification**

Pre-FAIRification pertains to the preparatory work before the actual implementation. Here, an inventory was made of everything necessary for developing and implementing the FAIR VASCA registry in a de novo manner. This includes objectives, team requirements, and resources (data, tools, budget).

***Step 1 - Identify FAIRification objectives and expertise***

First, the FAIRification objectives for the VASCA registry were identified based on current challenges in RD. The objectives help to set a scope for the FAIRification work to be done and to plan the FAIRification process. In short, these were to 1) base our VASCA registry on the CDEs and the FAIR principles to enable it for analyses across RD registries, and 2) implement de novo FAIRification in our VASCA registry, where data are made automatically FAIR upon collection.

Second, the expertise required to achieve the objectives were identified. Conducting the FAIRification process requires a highly multidisciplinary team guided by a FAIR data steward [1]. The VASCA FAIRification core team consisted of a local data steward, an external FAIR data steward, and an EDC system specialist. Throughout the project, additional expertise was consulted, such as a clinician specialised in vascular anomalies, the Institutional Ethical Review Board, FAIR software developers, and researchers. A full overview of the different kinds of expertise and which part of the FAIRification process they contributed to can be found in Table S1. Note, in our project, the expertise in many areas were provided by the same person. Also, research expertise is applicable throughout Table S1 and is for simplicity not specified. The areas of expertise have been learned from a previous project [1], and further advanced here.

***Step 2 - Define the data elements to be collected***

As defined in the FAIRification objective, the data collected in the VASCA registry were based on the CDEs [2]. The CDE ‘Classification of functioning/disability’ was not added, because there were many uncertainties about its use (see Discussion). We formally defined what data to collect for each of the other CDEs by interpreting the meaning of the CDEs and how they relate to each other. This was captured in an extensive manual for data collection. This task was done by the core FAIRification team together with clinicians specialised in vascular anomalies and a patient advocate. This was an important preparatory step for designing the eCRF (step 4) and creating the semantic data model (step 5) in the ‘facilitating FAIRification’ phase. This also contributes to consistent data entry by data managers across institutes, step 10 in the ‘data collection’ phase.

***Step 3 - Define the metadata elements to be collected***

This step entails identifying what metadata (description of data) should be collected (e.g., license, owner, contributions statements, and description of use conditions and access of data) to comply with the FAIR principles. The World Wide Web Consortium (W3C) Data Catalog Vocabulary (DCAT) [3] is the default standard to predefine and structure metadata elements in FAIR Data Points (as defined in the FAIR Data Point specification; see [4]). We decided to make the VASCA registry findable and accessible (under well-defined conditions) in a FAIR Data Point. Metadata elements described in the FAIR Data Point specification were therefore collected.

Metadata elements for the VASCA registry were also collected for ERDRI (ERDRI.dor and ERDIR.mdr). ERDRI.dor (European Directory of Registries) is a catalogue of RD registries. It contains the metadata related to the registry and includes 38 attributes, out of which 23 are compulsory. ERDRI.mdr (ERDRI Metadata Repository) contains detailed information about each variable collected in the registry including data type, description, and a list of permitted items.

**Phase II: Facilitating FAIRification**

In the second phase, the technical implementation in the EDC system was done to facilitate the de novo FAIRification. This pertains to e.g. the eCRF, the semantic data model, and the FAIR Data Point.

***Step 4 - Design the eCRF in the EDC system***

The eCRF was designed to collect data for the CDEs (described in step 2) in the Castor EDC system [5]. Several dependencies, e.g. only show ‘Date of death’ when the patient is deceased, and validations, e.g. validate whether the entered Online Mendelian Inheritance in Man (OMIM) genetic disorder code follows the OMIM standard, were included in order to collect high-quality data (the eCRF questions can be found in [6]). To this end, we mostly worked with closed questions and/or drop-down menus and prevented entering free text as much as possible. An example from the eCRF is shown in Figure S1A. The eCRF template containing the CDEs and the ontologies to annotate them (see step 5) was described in a codebook. This codebook was made openly available in ART-DECOR, a platform from Nictiz, the Dutch competence centre for electronic exchange of health and care information [7], and can be directly implemented in the Castor EDC system or other EDC systems using the openly available iCRF Generator tool [8].

***Step 5 - Create the semantic data model***

We created a semantic data model for the European Commission’s recommended set of CDEs to be used for the VASCA registry. The model is openly available on github [9]. A part of the model is shown in Figure S1B. First, our interpretations of the CDEs from step 2 were used to draw a conceptual model (listing the main concepts and relationships between the CDEs). This was done in close collaboration between the core FAIRification team, semantic data modelling experts and clinicians specialised in vascular anomalies to ensure that the intended meaning was captured. Later, machine processable ontologies were selected to replace the concepts in the conceptual model. A consequence of this process is that the representation of the data in the semantic data model is a product of our experts’ interpretations of the CDEs. Currently, the semantic data model of the CDEs is assessed and further optimised by the RD community (see Discussion).

The CDEs recommend diagnosis to be defined with the Orphanet Ontology (ORDO). However, the elements available in ORDO (ORPHAcodes) did not contain all terms in the ISSVA classification used clinically to classify diagnosis (i.e. some ISSVA terms were lacking in orpha). As a solution, we transformed the ISSVA classification into a machine-readable ontology and added mappings to ORDO. The more specific ISSVA terms not available in ORDO were mapped to more general available ORDO terms.

***Step 6 - Implement the semantic data model***

The semantic data model was implemented in a data transformation application in the EDC system, developed in this project (described in detail in [10]). An example of this implementation is shown in Figure S1C. Specific elements in the model that should be filled with eCRF data were marked as elements that require a value. Based on mappings between the eCRF and the semantic data model (see step 7 and Figure S1D), the application converts the entered eCRF data to a machine-readable RDF representation (see step 11 and Figure S1E) and stores it in a triple store (see step 13). RDF is used since we work with ontologised data in this project and it allows machine-readable representation of ontologies.

***Step 7 - Map the eCRF structure to the semantic data model***

The eCRF structure was mapped to the semantic data model implemented in the data transformation application (Figure S1D). Specifically, the eCRF questions were mapped to the elements in the semantic data model that require an eCRF value (i.e., an object in Figure S1C). The eCRF values are linked to ontology concepts that are used as a machine-readable representation of the value in the rendered RDF (Figure S1E). For example, the object ‘Status’ in the semantic data model (Figure S1C) is mapped to the eCRF question ‘Patient’s status’ and has a value of the eCRF that is an annotated value, which has one of the values listed under ‘Option’ in Figure S1D. If the patient’s status has the value ‘Alive’, the ontology concept ID PATO:0001421 (Figure S1D) will be added to the RDF (Figure S1E). The implemented semantic model can be reused in other databases that are built in the EDC system by creating a mapping between the semantic data model elements and the eCRF questions.

***Step 8 - Set up registry structure in the FAIR Data Point***

The available semantic metadata model of the FAIR Data Point specification was used to describe the VASCA registry [4]. This model is based on the DCAT standard. The VASCA registry FAIR Data Point metadata is described in three layers: 1) catalog - a collection of datasets, 2) dataset - a representation of an individual dataset in the collection, and 3) distribution - a representation of an accessible form of a dataset, e.g. a downloadable file or a web service that gives access to the data for authorised users (Figure S2). A catalog may have multiple datasets, and a dataset may have multiple distributions. The VASCA registry described in this project (Registry of Vascular Anomalies - Radboud university medical center) is one of the datasets in the catalog (Registry of Vascular Anomalies). Other VASCA registries, from this or one of the other centers can also be described in this catalog. The semantic metadata model of the FAIR Data Point metadata specification was implemented in the Castor EDC’s FAIR Data Point. The metadata that describe the catalog, dataset, and distributions of the VASCA registry described in this project, are publicly available and licensed under the CC0 license.

**Phase III: Data collection**

The third phase covers the actual collection of the clinical data including the process of obtaining informed consent.

**Step 9 - Obtain informed consent**

Our project was approved by the Radboud university medical center’s Institutional Ethical Review Board. Informed consent was obtained for each patient. The ERN template for obtaining informed consent was not used. Instead, custom made patient information sheets and informed consent forms were applied (see Discussion). Informed consent includes the use of the patient’s medical data for the VASCA registry as well as using this data in combination with data collected in other European registries or databases.

***Step 10 - Enter data in the eCRF***

Currently, data collection for the VASCA registry is a manual process, where data from the EHR is entered into the eCRF. Here, the symptoms described by the clinicians in natural language were manually converted into terms from HPO by using the HPO website [11].

The CDEs are static data elements, meaning that they do not include (changes over) time. Therefore, most data were collected and entered in the eCRF at the first contact in our centre. However, not all information is available at this point. For example, diagnostic imaging and genetic tests may still need to be performed. The results from these tests may provide new insights, thereby affecting CDEs such as genetic diagnosis, phenotype and age at which diagnosis was made. To include missing data or update data elements, we built in a six-month check, conducted six months after inclusion. At this point, data collected for the CDEs may be updated based on (newly) available information in the EHR.

**Phase IV: Generating FAIR data in real-time**

This phase entails the process of the actual de novo FAIRification of the VASCA registry. Here, the entered data and metadata are automatically converted into machine-readable representations. The machine-readable metadata constitutes the metadata in the FAIR Data Point. The machine-readable data is stored in a triple store (i.e., a specialised database to store and query RDF) and made available in the FAIR Data Point.

***Step 11 - Entered data is automatically transformed to RDF***

When the data is entered in the eCRF, it is automatically and in real-time converted into a machine-readable RDF representation by the data transformation application. Thus, the data is made machine-readable from the moment it is being collected: de novo FAIRification. This way, a periodic, manual conversion of the data into machine-readable language is not required, resulting in all data collected being available for reuse at any time. Also, updates in the semantic data model lead to automatic updates in the machine-readable RDF representations of data already collected. An additional benefit of this approach is that the people tasked with clinical care and data entry do not need this knowledge to generate FAIR data.

***Step 12 - Entered metadata is automatically transformed to RDF in the FAIR Data Point***

When the metadata is entered in the FAIR Data Point of the EDC system, it is represented in a human-readable format (a website, e.g. <https://fdp.castoredc.com/fdp/catalog/vasca>), and at the same time automatically converted into a machine-readable RDF representation, (e.g. the ttl format: <https://fdp.castoredc.com/fdp/catalog/vasca?format=ttl>).

***Step 13 - Store RDF data and make it available in the FAIR data point***

After transforming the eCRF data into a machine-readable RDF representation (step 11), it is stored in a triple store. This is done via the data transformation application upon data entry (collected or updated) in the EDC system (step 10). The URL providing access to the machine-readable data in the triple store is made available in the FAIR Data Point as an access URL in the Distribution layer (Figure S2).

**Phase V: Using FAIR data**

The final phase describes how the FAIR VASCA data available in the FAIR Data Point can be accessed and queried for research.

***Step 14 - Authentication and authorisation***

The VASCA registry metadata in the FAIR Data Point is open (CC0 license) and can be accessed by API calls. The actual registry patient data can only be accessed and queried by logging in with an authorised account of the EDC system (either viewing or exporting the RDF or querying the data using SPARQL). The process of providing access, authentication and authorisation, is currently arranged in the EDC system. Users (currently only humans) can request access to the data by contacting a specific contact person for the VASCA registry (a Data Catalog Vocabulary contact Point) provided in the metadata. Evaluating requests for access is currently a manual process and follows the permission given for sharing and exchanging data by the patient on the informed consent form (see step 9). The contact person has the authority to decide if access is granted or not. If access is granted an authorised account is provided to the user.

***Step 15 - Query over FAIR data point(s)***

The machine-readable data is stored in a triple store and can, therefore, be queried using the query language SPARQL by users with access to the data (described in step 14). Query results can be displayed in multiple formats (e.g. JSON, XML, CSV or TSV). The SPARQL endpoint of the EDC system can be queried by using external SPARQL clients or by using a web-based version that is available in Castor EDC’s FAIR Data Point. Currently, the web-based version can only query within a single database. Federated queries, therefore, need to be performed with external clients. These (federated) queries allow researchers to ask questions to the FAIR VASCA registry as well as other FAIR RD registries and data resources (multi-source analysis of FAIR data).

**References**

1. Jacobsen A, Kaliyaperumal R, da Silva Santos LOB, Mons B, Roos M and Thompson M. A Generic Workflow for the Data FAIRification Process. Data Intell. 2020; doi: 10.1162/dint_a_00028
2. European Commission, Set of Common Data Elements for Rare Disease Registration (CDEs), European Commission. https://eu-rd-platform.jrc.ec.europa.eu/set-of-common-data-elements_en. Accessed 4 Dec 2020.
3. D. Browning, DCAT 2 Vocabulary. https://www.w3.org/ns/dcat. Accessed: 4 Dec 2020.
4. FAIR Data Point. https://github.com/FAIRDataTeam/FAIRDataPoint-Spec. Accessed 4 Dec 2020.
5. Castor EDC, Castor Electronic Data Capture. https://castoredc.com. Accessed 4 Dec 2020.
6. Kersloot MG, Jacobsen A, Groenen KHJ, dos Santos Vieira B, Kaliyaperumal R, Abu-Hanna A, et al. The Joint Research Council’s Common Data Elements and their implementations on an electronic Case Report Form. figshare. 2021; doi: 10.1101/2020.12.12.20245951
7. VASCA Common Data Elements (CDE) - Datasets. https://decor.nictiz.nl/art-decor/decor-datasets--vasca-?id=&effectiveDate=&conceptId=&conceptEffectiveDate=. Accessed 4 Dec 2020.
8. The iCRF Generator. https://github.com/aderidder/iCRFGenerator. Accessed 4 Dec 2020.
9. Semantic data model of the set of common data elements for rare disease registration, LUMC. https://github.com/LUMC-BioSemantics/ERN-common-data-elements. Accessed 4 Dec 2020.
10. Kersloot MG, Jacobsen A, Groenen KHJ, dos Santos Vieira B, Kaliyaperumal R, Abu-Hanna A, et al. De-novo FAIRification via an Electronic Data Capture system by automated transformation of filled electronic Case Report Forms into machine-readable data. medRxiv 2021; doi: 10.1101/2021.03.04.21250752
11. The Human Phenotype Ontology. https://hpo.jax.org/app/. Accessed 4 Dec 2020.

**Supplementary table**

**Table S1.** **Expertise required for the FAIRification of a registry for vascular anomalies (VASCA).** Research expertise is not specified as it is applicable throughout the table. The areas of expertise are inspired from previous FAIRification projects*. Abbreviation: Electronic Data Capture (EDC).

|  | **What expertise is required? (modified from** [10]**)** | **Who provided this expertise**?** |
| --- | --- | --- |
| a | On the data to be FAIRified and how they are managed | - Local and FAIR data steward  - EDC system specialist  - Clinicians specialised in vascular anomalies  - Patient advocate for vascular anomalies |
| b | On the domain and the aims of the data resource within it | - Clinicians specialised in vascular anomalies  - Patient advocate for vascular anomalies |
| c | On architectural features of the software that is (or will be) used for managing the data | - EDC system specialist  - Software developer |
| d | On access policies applicable to the resource | - Local data steward  - Clinicians specialised in vascular anomalies  - Institutional Ethical Review Board |
| e | On the FAIRification process (guiding and monitoring it) | - Local and FAIR data stewards |
| f | On FAIR software services and their deployment | - EDC system specialist  - Software developer |
| g | On semantic data modelling | - Local and FAIR data steward  - Semantic data modelling specialists  - Clinicians specialised in vascular anomalies |
| h | On global standards applicable to the data resource | - Local and FAIR data stewards  - EDC system specialist  - Senior healthcare interoperability expert |
| i | On global standards for data access | - Local data and FAIR stewards  - EDC system specialist  - Senior expert of standards for automated access protocols and privacy preservation |

*** Jacobsen A, Kaliyaperumal R, da Silva Santos LOB, Mons B, Roos M and Thompson M. A Generic Workflow for the Data FAIRification Process. Data Intell. 2020; doi: 10.1162/dint_a_00028

*** Research expertise is applicable throughout the table and is for simplicity not specified.*

**Supplementary figures**


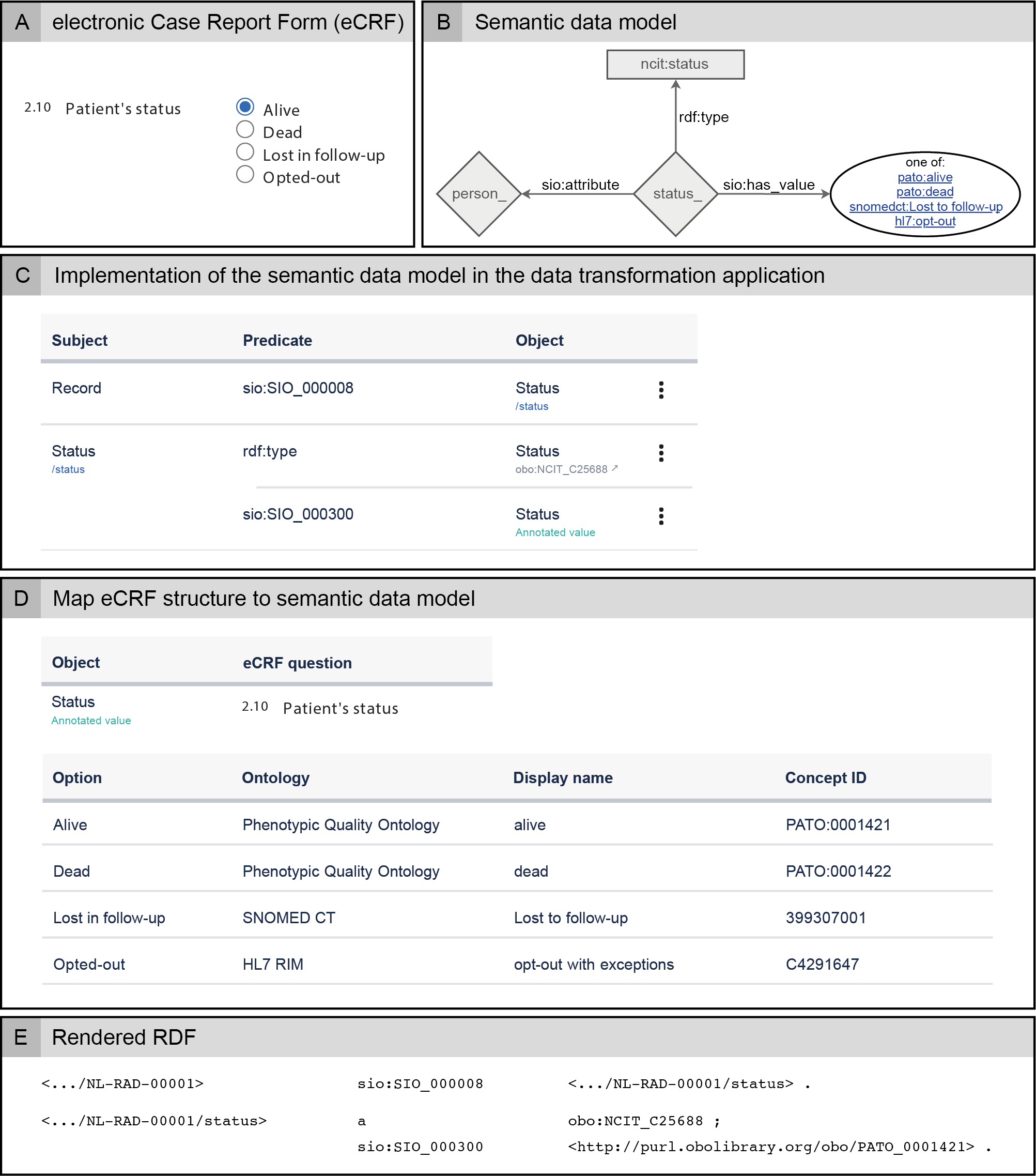


**Figure S1**. **Schematic representation of the generation of machine-readable data in the Resource Description Framework (RDF).** (E) The rendered RDF is based on (A) electronic Case Report Form (eCRF) data, (B) a semantic data model, (C) and the implementation of this model in a data transformation application and (D) mappings to the eCRF.


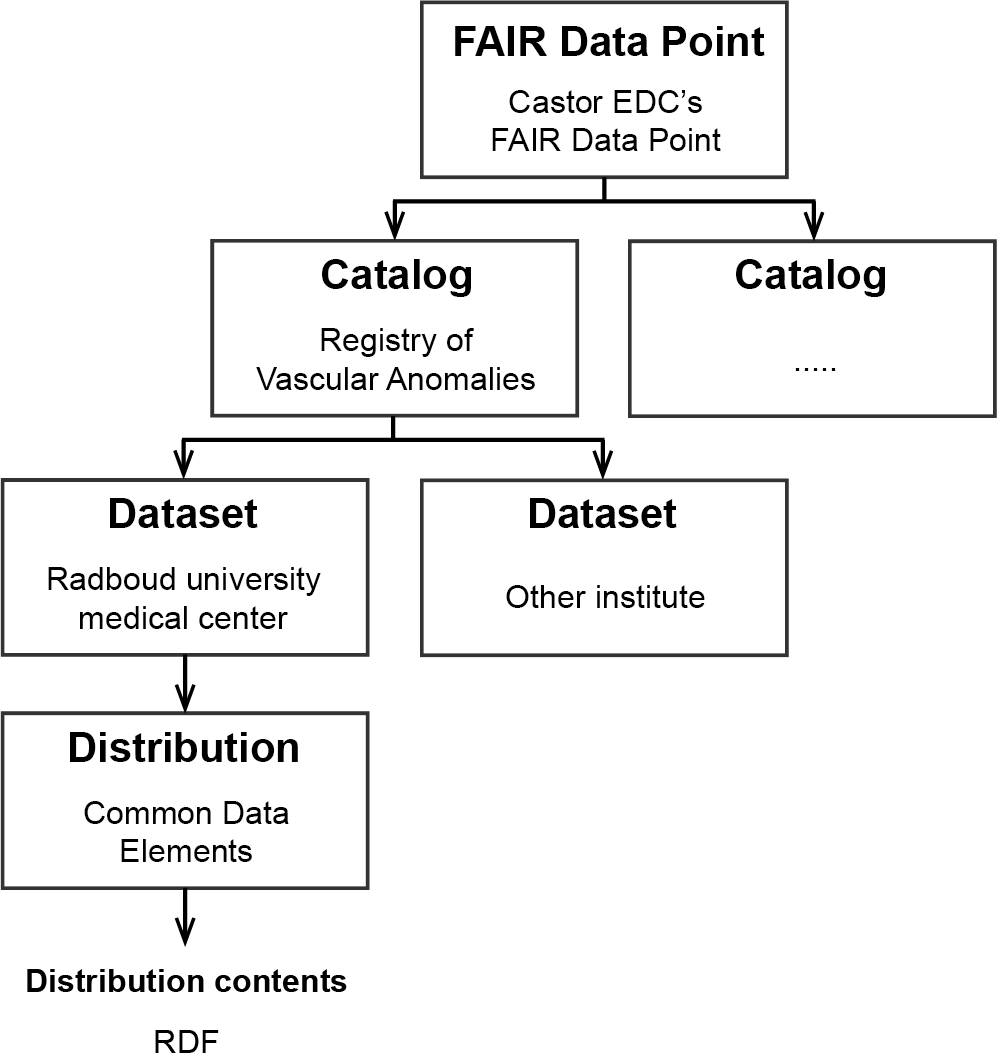


**Figure S2. Metadata layers for the Registry of Vascular Anomalies (VASCA) in Castor EDC’s FAIR Data Point.** It consists of three layers: Catalog, Dataset and Distribution. The Distribution layer connects a machine-readable representation of the clinical data collected in the Resource Description Framework (RDF), only for authorised users.
